# APTw CEST MRI in therapy-naive IDH-wildtype glioblastoma: insights into tumor heterogeneity and molecular subtypes

**DOI:** 10.1007/s11060-026-05616-1

**Published:** 2026-05-19

**Authors:** Thomas Zeyen, Andreas Decker, Inga Krause, Florian Kroh, Julia Scheuble, Lea L. Friker, Torsten Pietsch, Sebastian Regnery, Alexander Effland, Johannes Weller, Niklas Schäfer, Alexander Radbruch, Ulrich Herrlinger, Daniel Paech

**Affiliations:** 1 Department of Neurooncology, Center for Neurology and integrated Oncology (CIO), University Hospital Bonn, Bonn, Germany; 2 German Center for Neurodegenerative Diseases (DZNE), Bonn, Germany; 3 Department of Neuroradiology, University Hospital Bonn, Bonn, Germany; 4 Department of Radiology, Brigham and Women’s Hospital, Harvard Medical School, Boston, USA; 5 Institute of Neuropathology, University Hospital Bonn, Bonn, Germany; 6 Institute of Experimental Oncology, University Hospital Bonn, Bonn, Germany; 7 Department of Radiation Oncology, Heidelberg University Hospital, Heidelberg, Germany; 8 Institute for Applied Mathematics, University of Bonn, Bonn, Germany

**Keywords:** Glioblastoma, MRI, Amide proton transfer-weighted imaging, Chemical exchange saturation transfer imaging, DNA methylation, Mesenchymal subtype glioblastoma, Receptor tyrosine kinase subtype glioblastoma

## Abstract

**Purpose:**

Advanced MRI techniques may provide non-invasive insight into the molecular heterogeneity of glioblastoma. Amide proton transfer-weighted (APTw) chemical exchange saturation transfer (CEST) MRI reflects endogenous protein and peptide content, but its clinical and molecular correlates in therapy-naive glioblastoma, IDH-wildtype, remain incompletely understood.

**Methods:**

This retrospective single-center study included 53 adult patients with therapy-naive glioblastoma, IDH-wildtype, who underwent preoperative APTw MRI. Median time between imaging and tissue sampling was two days. Median and 90th percentile (p90) APTw signal intensities were extracted from contrast-enhancing (T1-CE) tumor regions and FLAIR-hyperintense regions using automated deep learning-based segmentation with manual quality control. Histological and molecular analyses included *MGMT* promoter methylation, Ki-67 index, and DNA methylation-based subclassification. Associations were assessed using non-parametric tests, multivariable linear regression, and Cox regression analyses.

**Results:**

APTw signal intensity was significantly higher in T1-CE tumor regions than in FLAIR-hyperintense regions (*p* < 0.0001). Within the T1-CE region, higher APTw signal intensity was modestly associated with younger age. Glioblastomas of the mesenchymal methylation subtype demonstrated significantly higher median and p90 APTw signal intensity compared with RTK1 and RTK2 subtypes, independent of *MGMT* status and Ki-67 index. APTw signal intensity was not independently associated with PFS or OS.

**Conclusion:**

APTw CEST MRI reflects molecular heterogeneity in therapy-naive IDH-wildtype glioblastoma, with potentially increased signal intensity in the mesenchymal subtype. These findings support its possible role as a complementary imaging biomarker for non-invasive molecular characterization.

**Supplementary Information:**

The online version contains supplementary material available at 10.1007/s11060-026-05616-1.

## Introduction

The diagnosis of brain tumors relies on histopathological and molecular analysis of tissue samples [1, 2]. However, magnetic resonance imaging (MRI) remains indispensable throughout clinical management - from preoperative planning to postoperative assessment and follow-up - providing noninvasive insights into tumor extent and response to treatment [3].

Conventional MRI is central to the differential diagnosis of intracranial lesions and to distinguishing gliomas from brain metastases [4, 5]. Typical features, such as the diffuse infiltration of gliomas versus the sharply demarcated, contrast-enhancing lesions of metastases, guide initial interpretation [6]. Yet, considerable overlap in imaging appearance persists, limiting diagnostic accuracy and complicating surgical and therapeutic decisions in some cases. Within the glioma spectrum, MRI features may also reflect underlying molecular profiles [7]. IDH-wildtype glioblastomas often present as contrast-enhancing lesions with central necrosis, whereas IDH-mutant astrocytomas appear more infiltrative and less enhancing [8]. Of note, imaging overlap is common, underscoring the need for more specific, biologically informative imaging biomarkers that could potentially facilitate a specific diagnosis based on non-invasive imaging techniques. Although tissue sampling and molecularly informed CNS tumor diagnosis is inevitably valuable, this might be especially important for cases where tissue sampling is considered a high-risk procedure, such as brain stem tumors or patients with comorbidities leading to a high risk of general anesthesia.

Advanced MRI techniques extend beyond morphology to probe tumor physiology and molecular composition [9, 10]. Among these, amide proton transfer-weighted (APTw) chemical exchange saturation transfer (CEST) MRI reflects endogenous protein and peptide content, providing indirect insight into tumor cellularity and metabolic state [11]. APTw imaging has shown promise in differentiating glioma grades and IDH mutation status [12–14], but data on its association with broader molecular and clinical characteristics remain limited. However, some studies are available investigating the association of APTw signal intensity with tumor characteristics other than IDH mutation status, including *MGMT* methylation status, ATRX status, p53 accumulation or the proliferation-index Ki-67 [12, 15, 16]. Notably, most of these studies consisted of heterogeneous patient cohorts regarding disease stage and prior therapies. This is especially important against the background of tumor heterogeneity and tumor changes upon treatment.

Methylation-based classification of gliomas has revealed that glioblastoma, IDH-wildtype, can be further subdivided into distinct molecular subgroups with potential prognostic and therapeutic relevance [17–19]. For instance, the mesenchymal subtype of glioblastoma shows limited benefit from extensive surgical resection compared to the receptor tyrosine kinase (RTK)1 or RTK2 subtypes [20].

This study investigated the APTw CEST MRI signal characteristics in therapy-naive glioblastoma with minimal time span from scanning to tissue analysis. It focuses on their associations with clinical parameters and molecular features, including methylation-based subtypes.

## Materials and methods

### Patient cohort and demographics

A total of 157 APTw MRI scans from adult patients with glioblastoma, IDH-wildtype, acquired between January 2020 and January 2023 during routine clinical imaging at the Neuro-Oncology Center Bonn, were retrospectively screened. Among these, 57 preoperative, therapy-naive APTw MRI scans were identified. Four scans were excluded due to technical issues (e.g., motion artifacts or reconstruction errors) identified during quality control. Consequently, 53 MRI scans from 53 individual patients were included in the final analysis (see supplemental Fig. 1). The MRI data used in this study have not been included in any previously published work.

### MRI and APTw imaging

MRI was performed on a 3 T Philips Achieva scanner (Best, Netherlands). The protocol included 3D-FLAIR, 3D-T1-weighted (before and after gadolinium - injection), and 3D-T2-weighted sequences. In accordance with consensus recommendations for clinical APTw imaging at 3 T, the 3D APTw sequence provided by Philips was used (turbo spin echo; voxel size = 1.8 × 1.8 × 6 mm³; FoV = 230 × 179.7 × 60 mm³; 10 slices; TE = 8.3 ms; TR = 6.1 s; B₁,rms = 2 µT; T_sat_= 2 s; duty cycle = 100%; 9 frequency offsets around ± 3.5 ppm and one M₀ reference; intrinsic B₀ correction; MTR asymmetry at + 3.5 ppm). Total acquisition time was 3 min 53 s. This protocol is consistent with recent recommendations and consensus guidelines on CEST imaging in brain tumors [21].

For each segmented region, the median and 90th percentile (p90) APTw signal intensities were extracted to reduce sensitivity to outliers. In the statistical analyses, the median values of these metrics across subjects were compared.

### Post-processing, registration and segmentation

Image post-processing and subsequent registration and segmentation was done as previously described [22]. The HD-GLIO deep learning algorithm enabled multimodal co-registration and automated 3D segmentation. For predominantly contrast-enhancing tumors, the algorithm generated non-overlapping segmentations of the contrast-enhancing tumor (T1-CE ROI) and the FLAIR-hyperintense peritumoral edema (FLAIR edema ROI), whereas for predominantly non-enhancing tumors, it generated a whole-tumor FLAIR ROI based on FLAIR hyperintensity (FLAIR non-CE ROI). Of note, in 6/53 (11.3%) no T1-CE ROI was available due to pre-dominantly non-enhancing tumors. All automated segmentations underwent visual quality control and were manually corrected if necessary (TZ and IK, each with > 3 years of neuroimaging experience) using MITK (version 2024.06.2). To avoid bias in APTw quantification, T1-weighted hypointense necrotic regions were carefully excluded from the ROIs. Similarly, cystic components and hemorrhages were excluded. The resulting T1-CE and FLAIR ROIs were then transferred to the corresponding APTw images.

### Tissue analysis, methylation profiling and NGS

Diagnosis of glioblastoma, IDH-wildtype (CNS WHO grade 4) was based on tissue sample analysis in all patients following WHO 2021 criteria [2]. Genomic DNA was extracted from FFPE tumor tissue, bisulfite-converted, and analysed using the Infinium Human MethylationEPIC BeadChip array (850 K/935K; Illumina) according to the manufacturer’s instructions. Methylation profiles were classified using the Heidelberg Brain Tumor Classifier (v12.8). *MGMT* promoter methylation status was assessed via Pyrosequencing as previously described [23].

### Statistical analysis

Statistical analyses were conducted using SPSS (version 29.0.0.0; IBM) and Prism (version 10.4.0; GraphPad Software). Descriptive statistics included frequencies and median values with corresponding ranges or interquartile ranges (IQR). For each ROI, voxel-based APTw signal values (median and 90th percentile, *p*90) are reported with their IQR. Group comparisons of APTw signal values were performed using the Mann-Whitney U test (comparison of two groups) or the Kruskal-Wallis test (comparison of more than two groups). Associations of APTw signal intensity with molecular markers were analysed via multivariable linear regression models. Pearson correlation analysis was performed to evaluate the association of age and APTw signal intensity.

Overall survival (OS) and progression-free survival (PFS) were estimated using the Kaplan Meier method and reported as median survival with 95% confidence intervals (CI). Numbers at risk are displayed below the Kaplan Meier curves. Cox regression analysis was performed to test for associations of APTw signal intensities and survival times.

For all statistical analyses, p-values < 0.05 were regarded as statistically significant. In the figures, significant results are marked as *≤0.05, **≤0.005, ***≤0.0005.

## Results

### Cohort, patient demographics and outcome

The cohort comprised 53 MRI scans from individual patients, all diagnosed with *Glioblastoma*,* IDH-wildtype*. The median age was 64 years (range: 38–87), and 64.2% were male (Table 1). The *O⁶*-methylguanine-DNA methyltransferase (*MGMT*) promoter was unmethylated in 30 of 53 cases (56.6%). Methylation subclassification data were available for 44 of 53 individuals (83%). Among these cases, 12 tumors were classified as RTK1, 19 as RTK2, and 7 as mesenchymal subtype. The remaining six patients either exhibited alternative subtypes or did not achieve a significant classifier match (score < 0.9) for any defined subclass. The majority of tumors were predominantly enhancing (86.8%), with only two cases showing bilateral hemispheric involvement; all others were unilateral. MRI demonstrated a cystic appearance in 8 of 53 patients (15.1%), including one tumor of the mesenchymal subtype, three of the RTK1 subtype, and four of the RTK2 subtype. Further details are summarized in Table 1. Median overall survival (OS) was 15.2 months (95% CI, 9.8–21.9), and median progression-free survival (PFS) was 6.7 months (95% CI, 6.0-7.8; see Supplemental Fig. 2). Median time between MRI and tissue sampling was two days (range: 1–23 days).

**Table 1:** Demographics, Baseline demographic, clinical, and molecular characteristics of the study cohort

Variable	*N* = 53
**Age (y)**	
Median (range)	64 (38–87)
**Sex**	
Male (%)	34 (64.2)
Female (%)	19 (35.8)
**Diagnosis**	
Glioblastoma, IDH-wildtype (%)	53 (100)
**MGMT status**	
MGMT-methylated (%)	22 (41.5)
MGMT-unmethylated (%)	30 (56.6)
N/A (%)	1 (1.9)
**Methylation subclass**	
RTK1 (%)	12 (22.6)
RTK2 (%)	19 (35.8)
Mesenchymal (%)	7 (13.3)
Other (%)	6 (11.3)
N/A (%)	9 (17.0)
**Contrast-enhancement**	
Predominantly enhancing (%)	46 (86.8)
Predominantly non-enhancing (%)	7 (13.2)
**Brain hemisphere**	
Right (%)	26 (49.1)
Left (%)	25 (47.2)
Both or corpus callosum (%)	2 (3.7)
**MRI appearance**	
Cystic (%)	8 (15.1)
Solid +/- necrosis (%)	45 (84.9)

Baseline characteristics of the 53 therapy-naive patients with glioblastoma, IDH-wildtype included in the final analysis. Continuous variables are reported as median (range), and categorical variables are reported as number (percentage). MGMT, O^6^-methylguanine-DNA methyltransferase; RTK, receptor tyrosine kinase; N/A, not available

### APTw signal intensity is highest in T1-CE region

**Fig. 1:**
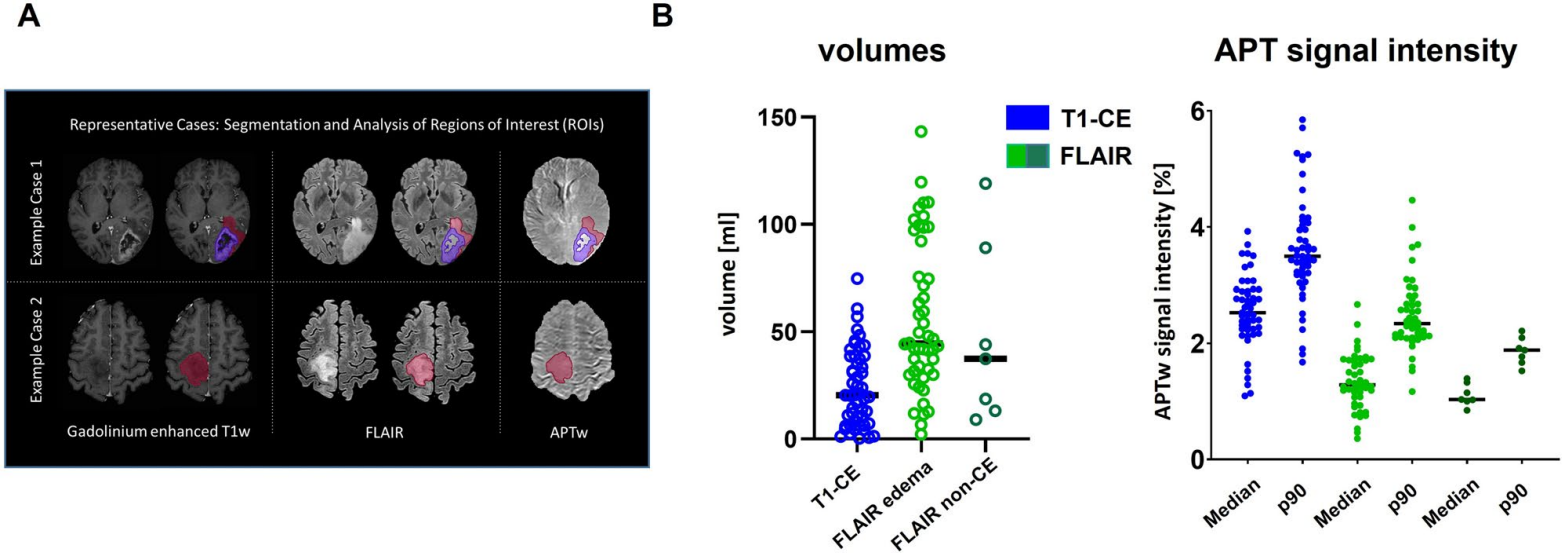
Tumor volumes and APTw signal intensity Tumor volumes and APTw signal characteristics in contrast-enhancing and FLAIR-defined regions. (**A**) Representative preoperative MRI examples demonstrating tumor delineation on post-contrast T1-weighted (T1-CE) and FLAIR images, with corresponding regions of interest used for volumetric and APTw signal analysis. (**B**) Scatter plots showing tumor volumes (left) and APT-weighted (APTw) signal intensity values (right) measured within T1-CE and FLAIR regions of interest. For APTw signal intensity, median and 90th percentile (p90) values are shown. Each dot represents an individual patient; horizontal lines indicate group medians

Illustrative examples of tumor ROIs (T1-CE and FLAIR) are shown in Fig. 1A. Mean volume of T1-CE ROI was 23.8 ml (± 18.0 ml) and mean volume of FLAIR edema ROI was 56.0 ml (± 34.5 ml, see Fig. 1B). Mean volume of FLAIR ROI in non-CE tumors was 43.4 ml (± 40.2 ml). Median APTw signal intensity in the T1-CE region was 2.5% (IQR: 2.2–2.9) with a median p90 of 3.5% (IQR: 3.2–4.1). In FLAIR edema median APTw signal intensity was 1.3% (IQR: 1.0–1.7) and median p90 was 2.3% (IQR: 2.1–2.7, see Fig. 1B). In non-CE tumors Median APTw signal intensity of FLAIR ROI was 1.1% (IQR: 1.0–1.4) and median p90 was 1.9% (IQR: 1.7–2.2).

### Highest APTw signal intensity was associated with glioblastoma of mesenchymal subtype

**Fig. 2:**
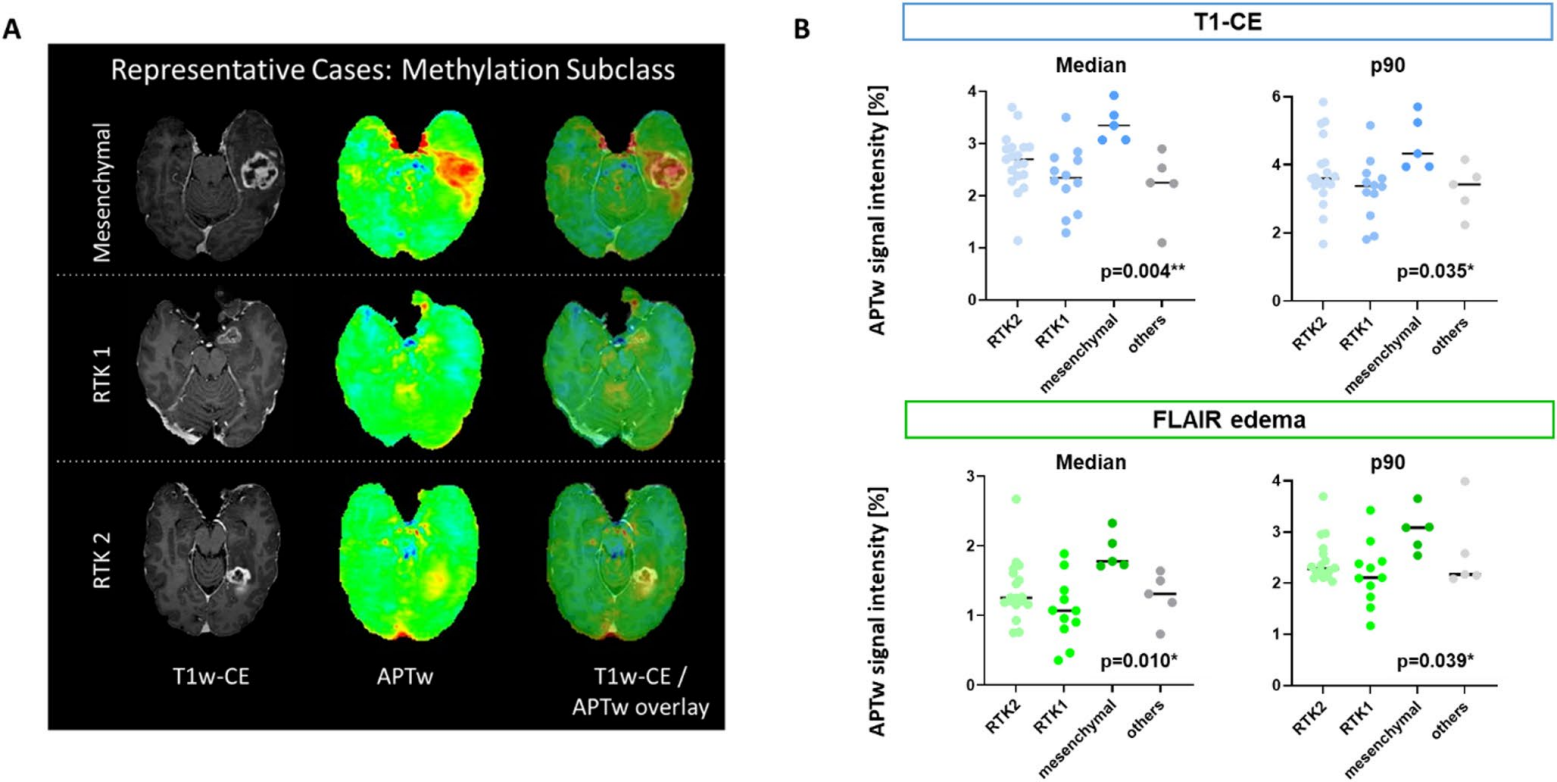
Association of APTw signal intensity with methylation subclass. APT-weighted signal intensity across DNA methylation subclasses. (**A**) Representative preoperative MRI examples demonstrating APTw signal intensities in different methylation subclasses. (**B**) Scatter plots showing APT-weighted (APTw) signal intensity within T1-weighted contrast-enhancing (T1-CE, top, blue) and FLAIR-defined (bottom, green) edema regions, stratified by DNA methylation subclass (RTK1, RTK2, mesenchymal, other). Median and 90th percentile (p90) APTw values are shown for each region of interest. Each dot represents an individual patient; horizontal jitter is applied for visualization

For subsequent analyses, only patients with CE-tumors on T1-weighted imaging (T1-CE; *n* = 46) were included to ensure comparability between T1-CE ROIs and FLAIR-defined edema ROIs. Median APTw signal intensity within the T1-CE ROI was comparable between glioblastomas of the RTK1 and RTK2 subtypes (2.34% [IQR 1.76–2.72] vs. 2.70% [IQR 2.38–2.93], respectively; see Fig. 2B). Notably, glioblastomas of the mesenchymal subtype exhibited a significantly higher median APTw signal intensity in T1-CE ROI (3.35% [IQR 3.07–3.73], *p* = 0.004; see Fig. 2B). Representative cases for each methylation subtype are shown in Fig. 2A. A similar difference was observed for the p90 value (*p* = 0.035). The same difference was found in FLAIR edema analysis (*p* = 0.01 for the median value and *p* = 0.039 for the p90 value). Supplemental Table 1 provides detailed values, including IQRs and ranges, for each methylation subclass and for non-CE tumors.

No significant differences in APTw signal intensity were observed with respect to MGMT promoter methylation status. However, MGMT-methylated glioblastomas showed a trend toward higher p90 values within the T1-CE ROI (3.76%) compared with MGMT-unmethylated tumors (3.46%; *p* = 0.08; see Table 2.2).

Similarly, no significant association was found between the Ki-67 index and APTw signal intensity. Nevertheless, tumors with higher Ki-67 values tended to exhibit higher median APTw signal intensity within T1-CE ROIs (2.69% vs. 2.34%; *p* = 0.16; see Table 2.1).

**Table 2:** Association of APTw signal with MGMT and Ki-67 index. Association of APT-weighted signal intensity with MGMT methylation status and Ki-67 index

Tumor feature	ROI	---	*P*-value
**Table 2.1**		**Median APTw signal (IQR; total range)**	
MGMT methylation status			
Unmethylated (*n* = 24)	T1-CE	2.48% (2.25–2.86; 1.10–3.50)	
Methylated (*n* = 21)	T1-CE	2.73% (2.32–3.07; 1.64–3.92)	0.183
Unmethylated (*n* = 24)	FLAIR edema	1.24% (0.93–1.68; 0.47–2.67)	
Methylated (*n* = 21)	FLAIR edema	1.37% (1.16–1.72; 0.36–2.33)	0.34
Ki-67 index			
Ki-67 < 15% (*n* = 14)	T1-CE	2.34% (2.22–2.78; 1.10–3.35)	
Ki-67 ≥ 15% (*n* = 32)	T1-CE	2.69% (2.30–2.93; 1.29–3.92)	0.16
Ki-67 < 15% (*n* = 14)	FLAIR edema	1.40% (1.04–1.74; 0.74–1.95)	
Ki-67 ≥ 15% (*n* = 32)	FLAIR edema	1.24% (1.10–1.70; 0.36–2.67)	0.45
**Table 2.2**		**P90 APTw signal (IQR; total range)**	
MGMT methylation status			
Unmethylated (*n* = 24)	T1-CE	3.46% (3.16–3.66; 1.9–5.16)	
Methylated (*n* = 21)	T1-CE	3.76% (3.31–5.06; 2.51–5.85)	0.08
Unmethylated (*n* = 24)	FLAIR edema	2.31% (2.09–2.88; 1.17–4.46)	
Methylated (*n* = 21)	FLAIR edema	2.36% (2.15–2.72; 1.53–3.65)	0.69
Ki-67 index			
Ki-67 < 15% (*n* = 14)	T1-CE	3.46% (3.02–3.76; 2.24–4.33)	
Ki-67 ≥ 15% (*n* = 32)	T1-CE	3.60% (3.24–4.15; 1.91–5.85)	0.28
Ki-67 < 15% (*n* = 14)	FLAIR edema	2.28% (2.13–2.82; 1.60–3.10)	
Ki-67 ≥ 15% (*n* = 32)	FLAIR edema	2.37% (2.11–2.73; 1.17–4.46)	0.75

Univariate analysis of APT-weighted (APTw) signal intensity according to MGMT promoter methylation status and Ki-67 proliferation index in therapy-naive glioblastoma, IDH-wildtype. Median and 90th percentile (p90) APTw values are reported for T1-weighted contrast-enhancing (T1-CE) and FLAIR-defined tumor regions. Data are presented as median (interquartile range; total range). Group comparisons were performed using the Mann-Whitney U test

In a multivariable linear regression model, the mesenchymal subtype was independently associated with higher median and p90 APTw signal intensity within the T1-CE ROI (Median: β = 0.81, *p* = 0.0026; p90: β = 0.93, *p* = 0.0319; see supplemental Table 2), whereas *MGMT* status and Ki-67 index were not significant predictors. In the multivariable analysis of the FLAIR ROI, similar patterns were observed, although the effect sizes were smaller.

### APTw signal intensity in therapy-naive glioblastoma is not associated with outcome

In univariable Cox regression analyses, higher APTw signal intensity within the T1-CE ROI was associated with longer PFS and OS (Tables 3.1 and 3.2). This association was observed especially for the p90 values. Specifically, the hazard ratio (HR) for PFS using the p90 T1-CE APTw signal was 0.64 (95% CI: 0.38–0.99), while the HR for OS was 0.40 (95% CI: 0.21–0.74). In contrast, APTw signal intensity measured within FLAIR ROIs showed no association with survival outcomes.

In multivariable Cox regression analyses adjusting for established prognostic factors - including age, extent of resection, *MGMT* methylation status, and therapy initiation (yes vs. no) – the p90 APTw signal intensity in the T1-CE ROI was no longer associated with PFS (HR: 0.89, 95% CI: 0.53–1.42; Table 3.3) or OS (HR: 0.59, 95% CI: 0.27–1.17; Table 3.4). In this model, *MGMT* methylation status, extent of resection, and therapy initiation remained strongly associated with survival outcomes.

**Table 3:** Cox hazard proportional regression

Variable	HR (95% CI)	*p*-value
**Table 3.1 PFS univariable**		
Median T1-CE P90 T1-CE Median FLAIR edema P90 FLAIR edema	0.51 (0.24–1.02) 0.64 (0.38–0.99) 1.02 (0.50–2.09) 0.86 (0.45–1.57)	0.06 0.049* 0.97 0.64
**Table 3.2 OS univariable**		
Median T1-CE P90 T1-CE Median FLAIR edema P90 FLAIR edema	0.28 (0.11–0.70) 0.40 (0.21–0.74) 0.82 (0.35–1.91) 0.75 (0.33–1.55)	0.006** 0.002** 0.65 0.45
**Table 3.3 PFS multivariable**		
P90 T1-CE Age MGMT [unmethylated] Extend of resection [no TR] First-line therapy [none]	0.89 (0.53–1.42) 1.01 (0.97–1.06) 3.50 (1.47–9.49) 1.58 (0.61–4.32) 66.9 (8.69–1382)	0.63 0.57 0.004** 0.35 < 0.001***
**Table 3.4 OS multivariable**		
P90 T1-CE Age MGMT [unmethylated] Extent of resection [no TR] First-line therapy [none]	0.59 (0.27–1.17) 1.01 (0.95–1.06) 3.29 (1.19–10.93) 4.53 (1.23–20.81) 53.45 (6.5–1203)	0.14 0.81 0.02* 0.02* < 0.001***

Cox hazard proportional regression Cox proportional hazards regression analyses evaluating the association between APT-weighted (APTw) imaging biomarkers and clinical variables with progression-free survival (PFS) and overall survival (OS). Hazard ratios (HR) are reported with 95% confidence intervals (CI). T1-CE, T1-weighted contrast-enhancing region; p90, 90th percentile

### APTw signal in glioblastoma is modestly associated with age, but not with sex

The median APTw signal intensity within the T1-CE ROI demonstrated a weak but statistically significant negative correlation with age, with slightly higher values observed in younger individuals (*r* = -0.38, *p* = 0.009; see supplemental Fig. 3A). A similar but weaker pattern was observed for the p90 APTw signal intensity (*r* = -0.30, *p* = 0.04; see supplemental Fig. 3A). In contrast, neither the median nor the p90 APTw signal intensity within the FLAIR ROI showed any meaningful association with age.

No associations were found between any APTw signal intensity metric and sex (see supplemental Fig. 3B). For example, the median APTw signal intensity in the T1-CE ROI was 2.57% in males and 2.50% in females (*p* = 0.74).

## Discussion

In this study, we investigated APTw CEST MRI signal characteristics in a homogeneous cohort of therapy-naive glioblastoma, IDH-wildtype patients with minimal delay between imaging and tissue sampling. Our results demonstrate that APTw signal intensity might be associated with patient age and distinct molecular features, most notably the mesenchymal methylation subtype, but does not independently predict progression-free or overall survival when established prognostic factors are taken into account.

### Regional differences in APTw signal intensity

Consistent with prior reports, APTw signal intensity was significantly higher in CE tumor regions than in FLAIR-hyperintense areas [24, 25]. This likely reflects the higher cellularity, protein content, and metabolic activity of viable tumor within the CE core, whereas FLAIR-hyperintense regions mainly represent edema, infiltrative margins, and reactive changes that dilute the tumor-specific signal. The absence of robust associations between FLAIR-based APTw metrics and clinical or molecular parameters in our cohort further suggests that APTw imaging primarily captures biologically relevant information within the solid tumor core rather than the peritumoral compartment. Notably, APTw signal intensity in FLAIR regions of non-contrast-enhancing tumors appeared similar to that in FLAIR edema ROIs, suggesting that blood-brain barrier disruption itself - reflected by contrast enhancement on T1-weighted MRI - may contribute to increased APTw signal intensity.

### APTw signal reflects molecular heterogeneity, particularly the mesenchymal subtype

One of the central findings of this study is the association between elevated APTw signal intensity and the mesenchymal methylation subtype of glioblastoma. Tumors of the mesenchymal subtype demonstrated significantly higher median and p90 APTw values in both contrast-enhancing and peritumoral FLAIR regions, an effect that remained significant in multivariable regression analyses adjusting for MGMT methylation status and proliferation index.

The exact biological determinants of increased APTw signal intensity in glioblastoma are not yet fully understood. Increased protein and peptide content within vital tumor tissue is considered the most likely explanation, although additional factors such as pH alterations, angiogenesis, and microenvironmental changes may also contribute [26–28]. Interestingly, while elevated APTw signal intensity has historically been associated with higher tumor grade, this relationship is not universal. For example, pilocytic astrocytoma, despite being a CNS WHO grade 1 glioma, can exhibit markedly elevated APTw signal intensity [29]. This has been attributed to high intracellular and extracellular matrix protein content as well as microcystic changes. Such observations suggest that APTw signal intensity may reflect specific tissue composition and tumor microenvironment characteristics rather than tumor grade alone.

In this context, the observation of higher APTw signal intensity in mesenchymal glioblastomas appears biologically plausible, as this subtype is characterized by a distinct tumor microenvironment. Mesenchymal tumors are associated with increased immune cell infiltration, inflammatory signaling, hypoxia, cellular stress responses, and extracellular matrix remodeling [30, 31]. These processes may be accompanied by altered protein expression and increased protein turnover, potentially contributing to elevated APTw signal intensity. APTw imaging may therefore provide a non-invasive imaging correlate of this molecular phenotype.

In contrast, RTK1 and RTK2 subtypes demonstrated largely overlapping APTw signal distributions, suggesting that APTw imaging may be more sensitive to broader transcriptional and microenvironmental differences, such as those characterizing mesenchymal tumors, than to receptor tyrosine kinase–driven molecular subgroups. These findings extend previous work that focused primarily on IDH mutation status and support the potential of APTw imaging to capture molecular heterogeneity within IDH-wildtype glioblastoma.

### MGMT, proliferation, and age effects on APTw MRI

*MGMT* promoter methylation was not associated with higher p90 APTw values in CE regions. Prior studies have reported inconsistent results, including higher APTw signal in *MGMT*-unmethylated tumors [15, 16] or no differences [12], likely due to cohort heterogeneity and treatment-related effects. Similarly, Ki-67 showed no significant association with APTw intensity despite a trend toward higher values in more proliferative tumors. Overall, these findings suggest that APTw signal reflects a combination of protein content, metabolic activity, and microenvironmental factors rather than MGMT status or proliferation alone.

We further observed a modest inverse association between APTw signal intensity in contrast-enhancing regions and patient age, with higher values in younger patients, while no association with sex was identified.

### APTw signal intensity in therapy-naive glioblastoma is not associated with outcome

In multivariable Cox regression analyses, APTw signal intensity within the CE tumor region was not associated with PFS or OS. Although this may seem inconsistent with prior reports linking higher APTw signal to worse survival, important methodological differences likely explain the discrepancy. Two of these studies assessed APTw imaging after radiotherapy, when treatment-related changes may influence signal intensity [32, 33]. The remaining study evaluated preoperative APTw MRI but included patients with low-grade gliomas, which typically exhibit lower APT signal and more favorable outcomes [34]. These differences in imaging time point and cohort composition likely account for the divergent findings.

These results indicate that while APTw imaging captures biologically meaningful tumor characteristics, it does not provide prognostic information beyond well-established clinical and molecular markers in therapy-naive glioblastoma. Rather than serving as an independent prognostic biomarker, APTw signal intensity may be better understood as a complementary imaging marker that reflects underlying tumor biology and molecular subtype. Although the mesenchymal subtype has been associated with poorer prognosis [35], the observed higher APTw signal intensity did not translate into prognostic significance in this cohort. This may be due to the small number of mesenchymal tumors (*n* = 7) and variability in subsequent treatment, including extent of resection and adjuvant therapy.

### Strengths and limitations

Key strengths of this study include the strict inclusion of therapy-naive patients, the short interval between imaging and tissue sampling, standardized APTw acquisition following consensus recommendations, and rigorous deep learning-based segmentation with manual quality control. These factors reduce confounding by treatment-induced changes and enhance the biological interpretability of the imaging findings.

Nevertheless, some limitations should be acknowledged. The retrospective, single-center design and moderate sample size - particularly for methylation subgroups - limit statistical power and generalizability. APTw imaging is also technically sensitive to factors such as B_1_ inhomogeneity, partial volume effects, and necrotic or cystic components, although careful exclusion of non-viable tissue was performed.

### Clinical implications and future directions

Our findings suggest that APTw CEST MRI is a potentially valuable imaging biomarker that provides non-invasive insight into molecular heterogeneity in glioblastoma, with potentially higher signal intensity observed in the mesenchymal subtype. It does not independently predict outcome in a homogeneous cohort of patients with therapy-naive glioblastoma, IDH-wildtype, after adjustment for established prognostic factors. Future studies should explore the integration of APTw imaging with other advanced MRI techniques and radiomic approaches to better delineate tumor biology and potentially support non-invasive molecular stratification. Longitudinal studies may further clarify how changes in APTw signal over time reflect treatment response or tumor evolution.

## Electronic Supplementary Material

Below is the link to the electronic supplementary material.

Supplementary Material 1

## Data Availability

The datasets generated during and/or analysed during the current study are available from the corresponding author on reasonable request.
