# Supplementary material for: APTw CEST MRI in therapy-naive IDH-wildtype glioblastoma: insights into tumor heterogeneity and molecular subtypes
Source: J Neurooncol. 2026 May 19;178(1):9. doi: 10.1007/s11060-026-05616-1 (PMC13186792; doi:10.1007/s11060-026-05616-1)
Supplement: Supplementary file 1 — Supplementary Material 1 [file 11060_2026_5616_MOESM1_ESM.docx]

**Supplementary material –**

**„ APTw CEST MRI in treatment-naive IDH Wildtype Glioblastoma is associated with mesenchymal subtype”**

**Supplemental figure 1 - flowchart for patient selection**

**
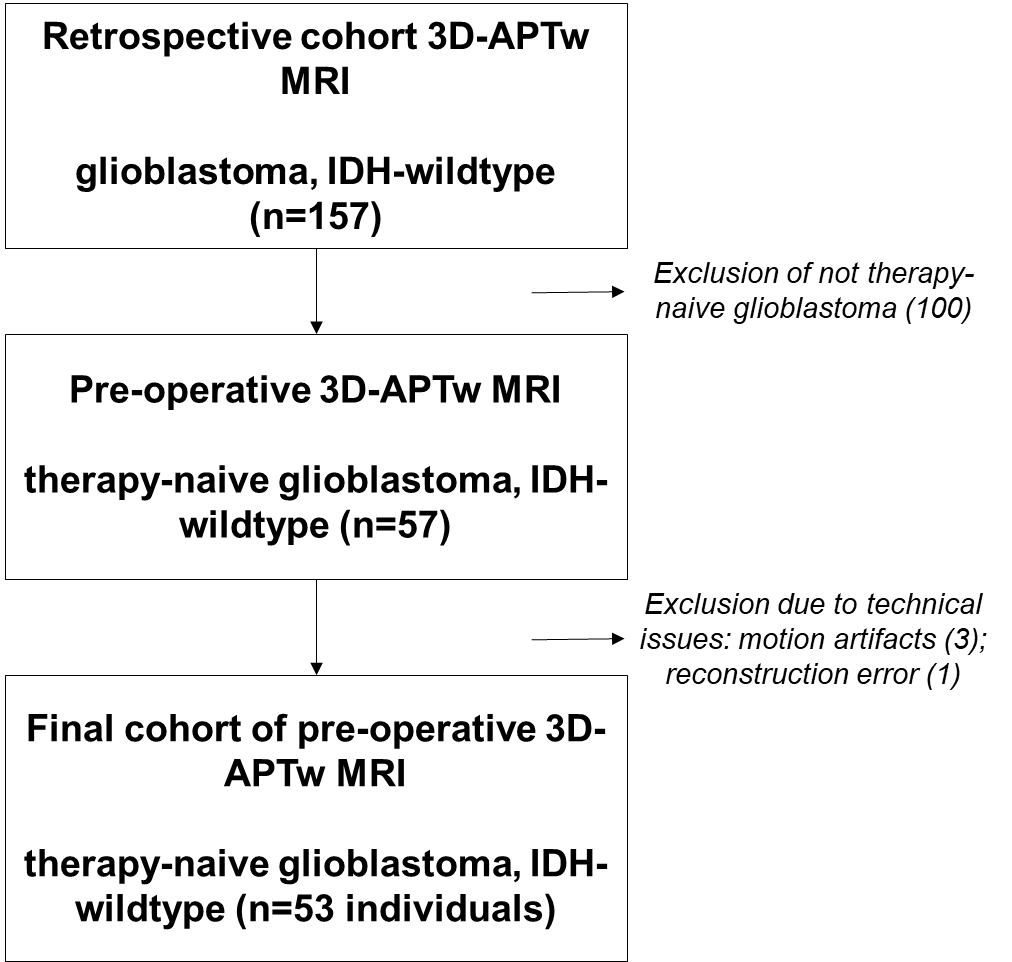
**

**Supplemental Figure 1. Patient selection flowchart.** Flowchart illustrating the selection of patients for the final study cohort. A retrospective cohort of patients with glioblastoma, IDH-wildtype who underwent 3D-APTw MRI (n = 157) was initially identified. Patients who were not treatment-naïve were excluded (n = 100), yielding a pre-operative, therapy-naïve cohort (n = 57). Of these, additional exclusions were made due to technical issues, including motion artifacts (n = 3) and reconstruction error (n = 1), resulting in a final cohort of 53 individuals included in the analysis.

**Supplemental figure 2 - outcome**

**
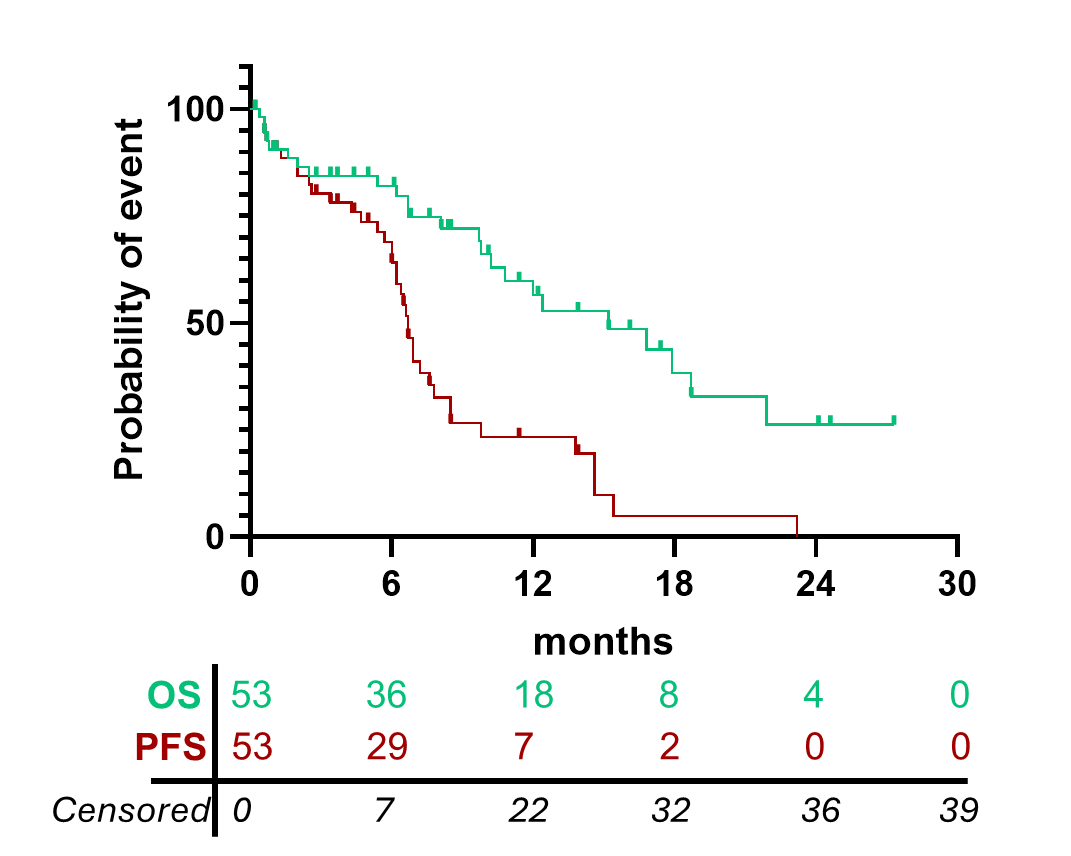
**

**Supplemental Figure 2. Kaplan–Meier survival curves for progression-free survival and overall survival.** Kaplan Meier estimates of progression-free survival (PFS, red) and overall survival (OS, green) in the final cohort of 53 patients with therapy-naïve glioblastoma, IDH-wildtype. Time is shown in months from the date of surgery. Tick marks indicate censored observations. Numbers at risk for OS and PFS at each time point are displayed below the plot.

**Supplemental figure 3 - association of APTw signal intensity with age and sex**


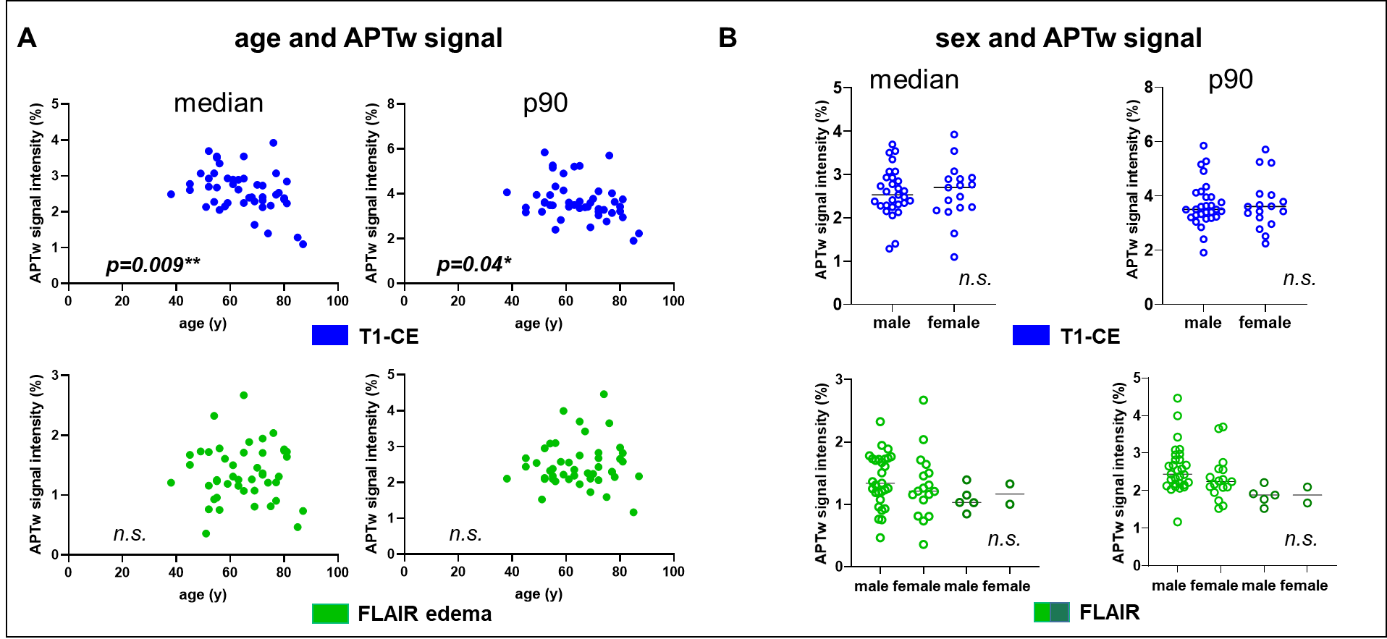


**Supplemental figure 3. Association of APTw signal intensity with age and sex.** (A) Scatter plots showing the relationship between patient age and APT-weighted (APTw) signal intensity within T1-weighted contrast-enhancing (T1-CE, blue) and FLAIR-defined (green) tumor regions. Median and 90th percentile (p90) APTw values are shown. A significant association between age and median APTw signal was observed in T1-CE regions (p = 0.03), while all other comparisons were not significant (n.s.). (B) Comparison of APTw signal intensity between male and female patients in T1-CE and FLAIR regions, shown for median and p90 values. No significant sex-related differences were observed.

**Supplemental table 1 - univariable analysis of APT-weighted signal intensity across DNA methylation subclasses.**

| Tumor feature | ROI | Median APTw signal (IQR; range) | P-value |
| --- | --- | --- | --- |
| Methylation subclass |  |  |  |
| RTK1 (n=19) | T1-CE | 2.34% (1.76-2.72; 1.29-3.50) |  |
| RTK2 (n=12) | T1-CE | 2.70% (2.38-2.93; 1.14-3.70) |  |
| Mesenchymal (n=5) | T1-CE | 3.35% (3.07-3.73; 3.07-3.92) | **0.0044** |
| Others (n=5) | T1-CE | 2.25% (1.67-2.71; 1.10-2.90) |  |
| RTK1 (n=19) | FLAIR edema | 1.07% (0.81-1.37; 0.36-1.89) |  |
| RTK2 (n=12) | FLAIR edema | 1.26% (1.16-1.63; 0.75-2.67) |  |
| Mesenchymal (n=5) | FLAIR edema | 1.78% (1.72-2.18; 1.71-2.33) | **0.0105** |
| Others (n=5) | FLAIR edema | 1.31% (0.96-1.57; 0.74-1.64) |  |
| RTK1 (n=1) | FLAIR non-CE | 1.004% (n.a.) |  |
| RTK2 (n=1) | FLAIR non-CE | 1.33% (n.a.) |  |
| Mesenchymal (n=2) | FLAIR non-CE | 1.15% and 1.03% (n.a.) | n.a. |
| Others (n=1) | FLAIR non-CE | 0.85% (n.a.) |  |

**Supplemental Table 1. Univariable analysis of APT-weighted signal intensity across DNA methylation subclasses.** Univariable analysis of APT-weighted (APTw) signal intensity stratified by DNA methylation subclass in treatment-naïve glioblastoma, IDH-wildtype. Median and 90th percentile (p90) APTw values are reported for T1-weighted contrast-enhancing (T1-CE) and FLAIR-defined tumor regions. Data are presented as median (interquartile range; range). Group comparisons were performed using the Kruskal-Wallis test.

**Supplemental table 2 - multivariable linear regression for APTw signal intensity in T1-CE ROI**

| Variable | value | β-estimate (95% CI) | P-value |
| --- | --- | --- | --- |
| Methylation subclass (mesenchymal) | Median | 0.81 (0.30–1.31) | **0.0026**** |
| MGMT (methylated) | Median | 0.18 (−0.17–0.54) | 0.3008 |
| Ki-67 (≥15%) | Median | 0.09 (−0.34–0.51) | 0.6796 |
| Methylation subclass (mesenchymal) | p90 | 0.93 (0.086–1.78) | **0.0319*** |
| MGMT methylated | p90 | 0.48 (-0.12–1.08) | 0.1112 |
| Ki-67 ≥15% | p90 | 0.21 (−0.5–0.93) | 0.5463 |

**Supplemental table 2. Multivariable linear regression for 90th percentile (p90) APTw signal intensity** Multivariable linear regression models evaluating the association between molecular and histopathological features and APT-weighted (APTw) signal intensity in the T1-weighted contrast-enhancing (T1-CE) tumor region. β-estimates are shown with 95% confidence intervals (CI). R² indicates the coefficient of determination for the full model.
